# Seizure-Induced Oxidative Stress in Temporal Lobe Epilepsy

**DOI:** 10.1155/2015/745613

**Published:** 2015-01-20

**Authors:** Sreekanth Puttachary, Shaunik Sharma, Sara Stark, Thimmasettappa Thippeswamy

**Affiliations:** Department of Biomedical Sciences, College of Veterinary Medicine, Iowa State University, Ames, IA 50011-1250, USA

## Abstract

An insult to the brain (such as the first seizure) causes excitotoxicity, neuroinflammation, and production of reactive oxygen/nitrogen species (ROS/RNS). ROS and RNS produced during status epilepticus (SE) overwhelm the mitochondrial natural antioxidant defense mechanism. This leads to mitochondrial dysfunction and damage to the mitochondrial DNA. This in turn affects synthesis of various enzyme complexes that are involved in electron transport chain. Resultant effects that occur during epileptogenesis include lipid peroxidation, reactive gliosis, hippocampal neurodegeneration, reorganization of neural networks, and hypersynchronicity. These factors predispose the brain to spontaneous recurrent seizures (SRS), which ultimately establish into temporal lobe epilepsy (TLE). This review discusses some of these issues. Though antiepileptic drugs (AEDs) are beneficial to control/suppress seizures, their long term usage has been shown to increase ROS/RNS in animal models and human patients. In established TLE, ROS/RNS are shown to be harmful as they can increase the susceptibility to SRS. Further, in this paper, we review briefly the data from animal models and human TLE patients on the adverse effects of antiepileptic medications and the plausible ameliorating effects of antioxidants as an adjunct therapy.

## 1. Introduction

Epilepsy is a serious neurological disorder manifested by recurrence of unprovoked seizures resulting in devastating effects on patients and the caregivers. The seizures are generated due to abnormal hypersynchronous paroxysmal cerebral discharges from the neurons which eventually results in irreversible damage to them and their surroundings. About 50% of reported cases of epilepsy are acquired [1]. The acquired causes such as head injury or infection or exposure to toxic chemicals can initiate one or more seizures or status epilepticus (SE) [2, 3]. Depending on the severity of the first insult, a varying period of latent period was reported during which a cascade of neurobiological changes takes place. These neurobiological changes culminate in the development of spontaneous recurrent seizures (SRS) resulting from synaptic reorganization into hyperexcitable and hypersynchronous neural networks [4]. According to International League Against Epilepsy (ILAE) multiple seizure episodes that occur within 24 hr are considered as a single event and hence SE is regarded as a single event. Established epilepsy refers to occurrence of two or more unprovoked recurrent seizures [2, 3]. A seizure occurring for a short duration is usually benign and self-limiting. Generalized convulsive SE is regarded as a clinical emergency due to significant morbidity and mortality [5]. Generalized convulsive SE in humans is attributed to continuous seizure lasting for 30 min or more consisting of two or more seizure episodes where the patient remains unconscious between the episodes [6, 7]. Considering the severe brain pathology associated with generalized convulsive SE, any seizure lasting for more than 5 min is treated as an emergency in clinics [6, 7]. It has been reported that some patients show nonconvulsive SE where EEG abnormalities are associated with impairment of consciousness that lasts at least 30 min without any obvious convulsive seizures [8]. The clinical signs of nonconvulsive SE are multifaceted exhibiting behavioral/cognitive changes such as confusion, agitation, hallucinations, facial automatisms with jerks, aphasia, nausea, pupillary abnormalities, and cardiorespiratory and thermal alterations [9]. Nonconvulsive SE is often underrecognized when compared to generalized convulsive SE [10]. The current antiepileptic drugs (AEDs) are merely symptomatic and do not prevent the progression of the disease. The greatest disadvantage with AED therapy is that its discontinuation makes the brain more vulnerable to the recurrent seizures and may get worse with time [11, 12].

In general, epilepsy afflicts more than 65 million people worldwide and over 100,000 new cases are added every year [13]. Among the epileptic patients, about 30% of them are refractory to the current AEDs [14]. Temporal lobe epilepsy (TLE) is one of the most common forms of partial or focal epilepsy which is associated with head traumas, brain malformations, infections, and febrile seizures [15]. In the United States alone over 3 million people suffer from epilepsy. In developing countries, the incidence is even higher due to a likelihood of cerebral infection in children during primitive obstetric services, head traumas in adults resulting from impacts, and a general susceptibility of elderly population to seizures. Severity of epilepsy depends on factors such as age, race, genetics, and socioeconomic and other environmental factors [13, 16]. The exact etiology of epilepsy is not well understood, but any kind of insult to the brain depending on its severity has a potential to induce seizures which can later develop into epilepsy. An alarming rise of epilepsy among different age groups, inconsistent cause and prognosis, morbidity, mortality, and above all its medically intractable nature in some of the patients make it of a top priority for research. Animal models have been instrumental in understanding the pathophysiology of epilepsy and for the preclinical studies for new drug discovery [17, 18]. In this review, we provide the information from animal models and human patients on the harmful role of ROS/RNS (reactive oxygen species/reactive nitrogen species) that are generated as a consequence to seizure and also discuss the role of gliosis, adverse effects of AEDs, and potential benefits of antioxidant supplements in TLE.

## 2. Oxidative Stress and Temporal Lobe Epilepsy

Studies have indicated that the loss of inhibitory neurons in the hippocampus during SE can alter the steady state of excitation and inhibition between neuronal populations towards hyperexcitability [19, 20]. This hyperexcitability initiates reactive gliosis and also results in mitochondrial dysfunction in neurons due to the generation of free radicals of oxygen and nitrogen species within the hippocampus and dentate gyrus. These changes will lead to neurodegeneration.

### 2.1. Free Radicals of Oxygen and Nitrogen Species

In normal physiological conditions, ROS and/or RNS levels are fairly well regulated to perform important functions such as autophagy, chemical signaling, cell division, and mitogen activated protein kinase signaling and apoptosis [21]. Due to the highly reactive nature of these molecules, the ROS and/or RNS are tightly regulated. Mitochondrial dysfunction due to ROS and RNS is frequently observed after seizures during epileptogenesis and is normally associated with neurodegeneration [22].

Free radicals contain one or more unpaired electrons in the outermost shell which confers them for being chemically reactive. Free radicals are generated by a loss of an electron or a gain of an electron during a homolytic cleavage [23, 24]. The resultant effect of homolytic cleavage is formation of two free radicals which may or may not carry an electric charge. Due to the presence of an excess electron or a lack of electron in their outermost orbits, these radicals behave as strong oxidants or reductants. Free radicals are highly unstable and reactive species, which initiate a chain reaction by pulling electrons from the nearby molecular fragments to form stable bonds, as a result the proteins and lipids will change their morphology and function. Such effects on DNA result in cross-linking of base pairs leading to mutation of a gene. Important free radicals of oxygen species include hydroxyl radical (OH^•^), superoxide anion (O_2_^−^), hydrogen peroxide (H_2_O_2_), singlet oxygen (O), alkoxy radical (RO), peroxy radical (ROO), and hypochlorite (HOCl). Widely known free radicals of nitrogen species include nitric oxide radical (NO^•^), peroxynitrite radical (ONOO^−^), nitroxyl anion HNO^−^, nitrosonium cation (NO^+^), higher oxides of nitrogen (N_2_O_3_, NO_2_^•^), and S-nitrosothiols (RSNO) [25–28]. The production of these radicals within the cell in excessive amount can lead to oxidative stress.

### 2.2. Free Radical Production and Oxidative Stress

An oxidative stress generally refers to a biochemical state where ROS or RNS production is unregulated resulting in damage to the cell membrane, proteins, enzymes, and DNA components within the nucleus and the mitochondria [24]. A majority of RNS are generated from the interactions of nitric oxide (NO) and oxygen. NO is an important second messenger, which can also behave like a free radical due to the presence of an unpaired electron in the outermost orbit (6 valence electrons from oxygen and 5 from nitrogen) [28]. NO is produced from the substrate, L-arginine via the enzyme NO synthase (NOS) involving nicotinamide adenine dinucleotide phosphate (NADPH) and oxygen. There are three major isoforms of NOS: (a) neuronal NOS (nNOS) produced by neurons, (b) endothelial NOS (eNOS) expressed mainly endothelial cells, and (c) inducible NOS (iNOS) induced in immune cells, astrocytes, microglia, and also neurons. The roles performed by NO vary based on its synthesis from the NOS isoforms and the tissues in which it is produced [29, 30]. The physiological concentrations of NO produced by nNOS mediate calcium dependent protein modification (S-nitrosylation), energy metabolism (through cytochrome C oxidase), synaptic plasticity, and neuroprotection. The NO produced by eNOS results in calcium dependent cyclic guanosine monophosphate (cGMP) mediated vasodilation to maintain vascular tone of cerebral blood vessels. The NO by iNOS is important for immune response or killing pathogens by generating free radicals [31–37]. However, excessive amount of NO produced by iNOS-mediated mechanism is harmful to the host cells.

**Figure 1:**
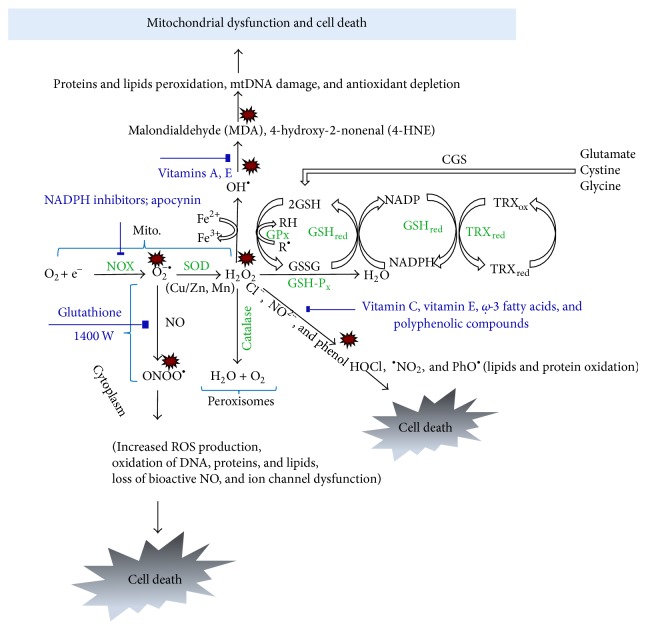
Biochemical reactions of ROS/RNS and their elimination by cellular endogenous antioxidants. Components in blue represent nonenzymatic antioxidants; green represents oxidative and antioxidant enzymes; and small red explosion sign represents generation of free radicals. NOX is the key enzymatic source of ROS. It reduces oxygen to superoxide anion and hydrogen peroxide. O_2_^•^ forms H_2_O_2_ which is the most reactive radical among its group that is produced via Fenton reaction. OH^•^ leads to lipid peroxidation by producing harmful metabolites such as MDA and 4-HNE leading to mitochondrial dysfunction and cell death. It also produces HOCl^•^ and PhO^•^ which are extremely toxic oxidants that disrupt tight junctions and increase paracellular permeability. H_2_O_2_ is eliminated by CAT, in peroxisomes, and GPx (location varies). At rapid rates, superoxide anions compete with NO which results in the formation of highly reactive molecule called peroxynitrite (ONOO^•^), in cytoplasm, leading to increased ROS production, oxidation of DNA, RNA, and proteins, ion channel dysfunction, and loss of bioactive NO^•^. Peroxynitrite inactivates Mn-SOD, thereby increasing the flux of superoxide anions available to react with NO. SOD catalyzes the reduction of superoxide anions into H_2_O_2_, in mitochondria in the presence of enzymes GPx and CAT; H_2_O_2_ gets converted into water and oxygen. Antioxidant enzymes such as GPx oxidize GSH to GSSG and GSH_red_ recycles GSH back from GSSG. NADPH gets reduced to NADP by GSH_red_. GSH/GSSG is a commonly used biomarker of oxidative stress in biological systems. However, GPx also catalyzes H_2_O_2_ into H_2_O by using reduced TRX_red_. Antioxidant defense against toxic oxygen intermediates comprises an intricate network which is heavily influenced by nutrition (vitamins A, E, and C and fatty acids). CGS plays an important role in glutathione metabolism and acts as an antioxidant in glial cells such as astrocytes. Extracellular oxidized cysteine is reduced to cysteine by thioredoxin reductase or glutathione that helps to maintain the steady state balance between antioxidants and ROS [24, 41, 80]. ROS, reactive oxygen species; NADPH, nicotinamide adenine dinucleotide phosphate; NOX, NADPH oxidase; SOD, superoxide dismutase (Cu/Zn—copper/zinc, Mn—manganese); CAT, catalase; O_2_^−•^, superoxide anion; H_2_O_2_, hydrogen peroxide; NO, nitric oxide; ONOO^−^, peroxynitrite; HOCl, hypochlorous acid; PhO^•^, phenoxy radical; OH^•^, hydroxyl radical; GSH, glutathione; GSSG, oxidized glutathione; TRXox/red, thioredoxin reduced and oxidized; TRX_red_, thioredoxin reductase; GSH_red_, glutathione reductase; GPx, glutathione peroxidase; CGS, cystine/glutamate antiporter system.

Generation of free radicals under normal conditions within a cell is depicted in Figure 1. In the cytoplasmic membrane, NADPH oxidase (NOX) reduces O_2_ to superoxide anion (O_2_^−^). Superoxides can also be generated from O_2_ from xanthine oxidase during the production of uric acid. These superoxides are converted into H_2_O_2_ in the presence of superoxide dismutase (SOD). H_2_O_2_ is a lipophilic molecule, which crosses lipid membranes into peroxisomes where it is finally eliminated by catalase (CAT) releasing H_2_O and O_2_ [38]. However, if the antioxidant action of SODs or CATs is impaired then, the reaction of superoxide with H_2_O_2_ yields toxic OH radicals in the presence of Fe^2+^ (called Fenton and Haber-Weiss reaction). These OH radicals can also be generated by superoxides when they react with hypochlorite (HOCl) [24, 38]. The hypochlorite (HOCl) arises when chloride (Cl^−^) reacts with H_2_O_2_ catalyzed by peroxidases. The OH radical is a harmful free radical of oxygen which has a short half-life but remains highly reactive. Further, the hydroxyl radical can also react with NO to form peroxinitrate (ONOO^−^), a powerful oxidizing agent that can cause lipid peroxidation, tyrosine nitration, and cytotoxicity [24, 27, 39].

Besides the major pathways of free radical production, other enzymes and pathways can contribute to excessive accumulation of ROS/RNS in cells. While they are not the primary sources of ROS/RNS, these enzymes and pathways are capable of accelerating the process of neurodegeneration. NOX also mediates the production of superoxide radicals in the hippocampus. At basal level, these play a role in learning and memory consolidation [40, 41]. However, under pathological conditions such as TLE, NOX overproduces superoxide ions to initiate neurodegeneration. Hence, the compounds that inhibit NOX enzymes could be beneficial in the treatment of epilepsy. A review by Sorce and colleagues describes the advantages of inhibiting of NOX during reactive gliosis and neuronal injury in rat models [42, 43]. In addition to NOX, the cyclooxygenase-2 (COX-2) enzymes have been found to upregulate ROS levels via the production of prostaglandins (specifically, F_2_ and H) [44]. In an* in vitro* model of rat cortex, it has been shown that the prostaglandins stimulate astrocytes to produce proinflammatory cytokines, which initiated neuronal death [45]. COX-2 is also responsible for a number of inflammatory responses in tissues involving neutrophils of the immune system [46]. COX-2 inhibition prevented lipid peroxidation within the mice brain and hence COX-2 could be another potential drug target for epilepsy [44, 47].

### 2.3. Free Radical Neutralization by Endogenous Antioxidant System

Cells possess native antioxidant systems to neutralize free radicals when produced in excess [68]. In the cytoplasm, SOD enzyme is coupled to copper and zinc ions (Cu-Zn SOD, also known as SOD-1), and, in the mitochondria, it is coupled to manganese (Mn-SOD, also known as SOD-2). SOD is an important antioxidant enzyme that scavenges superoxide radicals by catalyzing them into water and molecular oxygen. SOD-1 levels were low in cerebrospinal fluids of human patients with refractory epilepsy [69] suggesting that low levels of SOD-1 increase ROS. Intravenous administration of SOD-1 increased the seizure threshold in amygdala kindling rat models of epilepsy [70]. Experiments with SOD-2 knockout mice have been shown to be susceptible to kainate induced neurodegeneration and neuronal cell death [71].

The fate of H_2_O_2_ for conversion into H_2_O and O_2_ is determined by CAT enzymes (in mitochondria and peroxisomes), glutathione peroxidase (GSH-Px, in cytosol and also found extracellularly combined to selenium), and glutathione-S-transferase (GST, in cytosol and microsomes) [72]. The reactions include utilization of reduced glutathione (GSH) to combine with H_2_O_2_ to form H_2_O to release oxidized glutathione (GSSG) [73, 74]. Thus availability of reduced GSH becomes an important antioxidant reserve of the cell. The reduced GSH is resynthesized from GSSG by glutathione reductase (GSH_red_) utilizing NADPH. The NADPH for this process is generated by thioredoxin reductase (TRX_red_) found in endoplasmic reticulum by utilizing oxidized thioredoxin (TRX_ox  _) [75–77]. In addition to the antioxidant enzymes, peroxiredoxins, a ubiquitous family of antioxidant enzymes, degrade H_2_O_2_ and peroxynitrites to H_2_O and nitrites [78, 79].

### 2.4. Susceptibility of Brain to Oxidative Stress

While brain accounts for about 2% of body weight, it consumes 20% of the total inspired oxygen at rest [89]. This is due to a high metabolic rate of the neurons and the need for large amounts of ATP to maintain ionic gradient to sustain normal neurotransmission. Hence, mitochondria are found abundant in neurons' synaptic terminals to supply ATP, which is generated through oxidative phosphorylation [90]. In mitochondria, during normal oxidative phosphorylation, free radicals are also generated in small quantities from electron transport chain (ETC) complexes 1 and 3 [91]. In addition, brain contains large amounts of readily oxidizable polyunsaturated fatty acids which are necessary for the lipid membrane's structure and function. During oxidative stress, polyunsaturated fatty acids become susceptible to lipid peroxidation. This affects the permeability of the membrane to ions and signal transduction [92, 93]. Further, neurons are also rich source of iron, an important element in many cellular processes and physiological functions. During oxidative stress, high amounts of iron can prove harmful as iron participates in the redox reactions to generate ROS via Fenton and Haber-Weiss reaction [94]. Furthermore, CAT enzyme levels (essential for the cleavage of H_2_O_2_) are low in the brain compared to other organs, for example, 1/10th of liver CAT activity, making it susceptible to oxidative damage [38, 39, 95, 96]. However, under normal conditions, the innate antioxidant systems provide antioxidant protection against the ROS/RNS damage during metabolic processes [97, 98].

## 3. Seizure Insult Increases Oxidative Stress

**Figure 2:**
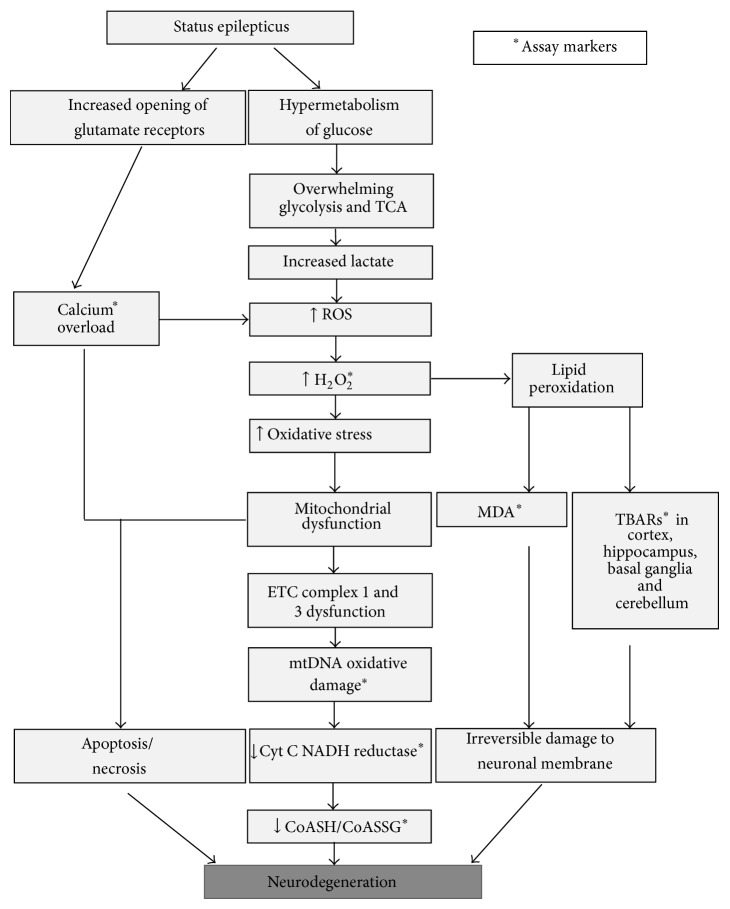
Post-SE pathways in neurodegeneration. SE increases glutamate receptors subunits interactions (NMDA, AMPA, and metabotropic), receptor turn-over, and their trafficking to the postsynaptic membrane. This leads to rapid calcium influx and calcium overload. As a result of this, several calcium dependent enzymes get activated in uncontrolled manner. This results in the activation of several signaling pathways that causes mitochondrial swelling, decrease in ATP, and increase in ROS, which results in oxidization of protein, lipid, and DNA, causing neuronal death. In addition, hypermetabolism, overwhelming glycolysis, and TCA cycle during SE further increase ROS/RNS. High production of lactate can cause cerebral lactic acidosis thereby increasing the production of ROS causing further damage due to mitochondrial dysfunction. Excessive calcium and ROS leads to the collapse of mitochondrial membrane potential, activation of mitochondrial matrix enzymes, and opening of mitochondrial permeability transition pores, decreasing ATP production. ROS are produced in mitochondria through the activity of ETC as a by-product of oxidative phosphorylation. CoASH/CoASSG and GSH/GSSG (described in Figure 3) ratio also decrease in brain tissues during this process and following SE, due to increased oxidative stress [44, 81–84]. TCA: tricarboxylic acid cycle; ETC: electron transport chain; mtDNA: mitochondrial DNA; Cyt C NAD: cytochrome NADH reductase; CoASH: coenzyme A; CoASSG: coenzyme A glutathione disulfide; SE: status epilepticus.

Oxidative stress and mitochondrial dysfunction have been long recognized as key mechanisms in several neurological disorders. Emerging evidence confirms that oxidative stress manifests as a consequence of the first seizure insult, which turns out later to become the cause of epileptogenesis [99]. During brain injury that results from seizures in rodent models, a significant increase in neuronal glucose uptake and metabolism was observed [81, 100]. Cerebral blood flow is found to increase in order to cope with hypermetabolism of glucose, thus resulting in buildup of lactate, thus overwhelming the normal glycolysis and tricarboxylic acid (TCA) cycle. The recurrent seizures can also result in overproduction of mitochondrial superoxide radicals in rodent models [48] that can be converted to hydroxyl radical via Fenton and Haber-Weiss reaction. The hydroxyl radical in the presence of Cu^2+^ and Fe^2+^ ions readily oxidizes proteins, lipids, and DNA resulting in altered protein function, membrane permeability, and gene expression, respectively. These events increase neuronal excitability and also decrease seizure threshold. Several lines of evidence showing the link between oxidative stress and the mitochondrial dysfunction due to seizures have been observed in human patients and rodent models of TLE (Table 1; Figures 2 and 4). Briefly, it is summarized here.

- An increase in calcium overload due to excitotoxicity and increased ROS production during seizures predisposing neurons to degeneration [101–103]. There is an increased oxidation of macromolecules of the neurons after SE prior to the neuronal loss [104, 105].
- Presence of neuronal death predominantly in CA3 and/CA1 regions of hippocampus following the first seizure [106], thus the TLE. Another example for CA1 hippocampal neurodegeneration is shown in Figure 4 (7 days after seizure).
- Changes in the mitochondrial membrane potential and increased NADPH levels as a consequence to seizures in rodent models and human patients [107].
- A significant increase in neuronal glucose uptake and enhanced metabolism in brain following the first seizure [81, 100].
- Inactivation of mitochondrial aconitase levels after SE [48].
- A reduction of mitochondrial N-acetyl aspartate (a metabolite synthesized from aspartate and acetyl-coenzyme A) in hippocampus from human epileptic patients [108–110].
- Dysfunctional electron transport chain complexes (1, 3, and 4) after SE [82, 111–113].
- A rise in mitochondrial H_2_O_2_ production, lipid peroxidation (increased malondialdehyde, MDA, and thiobarbituric acid, TBA), and mitochondrial DNA (mtDNA) damage following a seizure [58, 85, 114–116].
- An increase in seizure susceptibility in aging mice and/or SOD mice due to compromised innate antioxidant mechanisms [117, 118].
- NMDA receptor antagonists [119] and antioxidant supplements (SOD mimetics, vitamin C, vitamin E, and melatonin) administration preventing seizure-induced neuronal death [120–124].

## 4. Oxidative Stress Increases Hyperexcitability during Epileptogenesis 

**Figure 3:**
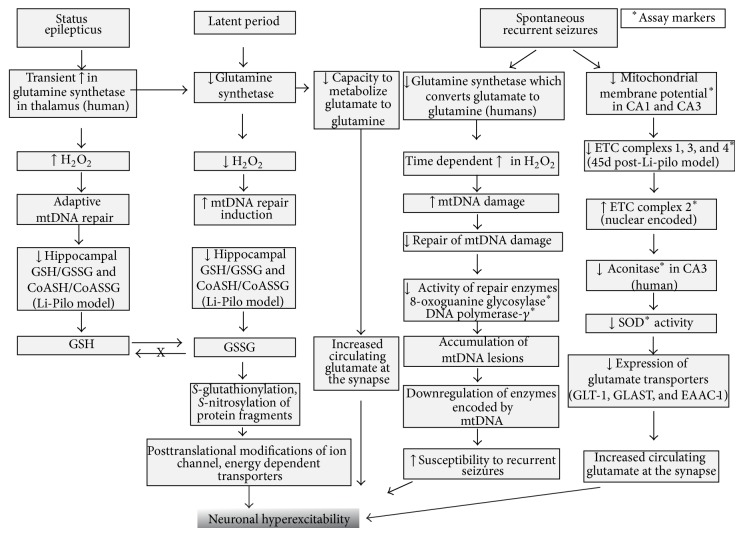
Sequence of events during status epilepticus, latent period, and SRS. The damage to the specific areas of the brain during SE can initiate varying period of neurobiological changes that can lead to the development of SRS. The enzymes free radicals and the pathways involved in these disorders are common in all types of insult (SE, latent period, and SRS), as described in Figures 1 and 2, but with subtle differences. The concentration of antioxidant enzymes rises after an initial insult (imitating their protective role) such as glutamine synthetase in SE; later it reduces which may or may not recover after few days/weeks depending upon the severity of the insult. Latent period is generally characterized by a series of slow neurodegenerative changes in the brain leading to epileptogenesis. The concentration of GST falls during latency that affects glutamate metabolism. High levels of the glutamate in the extracellular and intracellular space can lead to neuronal excitability through activated calcium signaling, as described in Figure 2. Levels of H_2_O_2_ return to the basal levels with mtDNA repair and low GSH/GSSG and CoASH/CoASSG ratio. During latent period, nitrosylation of protein fragments and posttranslational modifications of ion channels and transporters will further lead to hyperexcitability of neurons [24, 48, 82, 83, 85, 86]. SRS: spontaneous recurrent seizures; GST: glutamine synthetase; mtDNA: mitochondrial DNA; X: reverse reaction does not occur; Li-Pilo: lithium- pilocarpine model.

**Figure 4:**
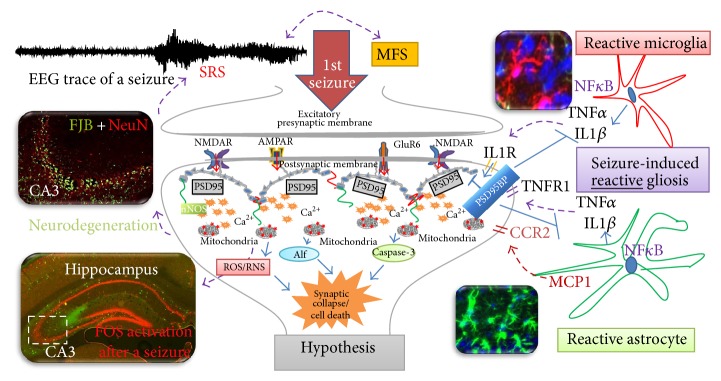
Schematic representation of a synapse, with postsynaptic ionotropic glutamate receptors (NMDA, AMPA, and KA/GLUR6), its associated glial cells, and extrasynaptic effects of a seizure. First seizure due to hyperexcitability of neurons (as evident from increased Fos expression in the hippocampus) induces reactive gliosis at a later stage, which produces inflammatory cytokines and iNOS that are mediated by NF*κ*B transcription. These in turn sensitize postsynaptic neurons and decrease their seizure threshold. Reactive astrocytes also downregulate glutamate uptake, thus increasing the concentration of glutamate at the synapse. These events contribute to further hyperexcitability of neurons as evident from increased spiking activity on EEG. These changes in turn lead to neurodegenerative changes after 3 days following the first seizure (Fluoro-Jade-B (FJB)+, neuronal nuclei protein (NeuN), the markers used to detect neurodegeneration) [30, 35, 87, 88].

**Figure 5:**
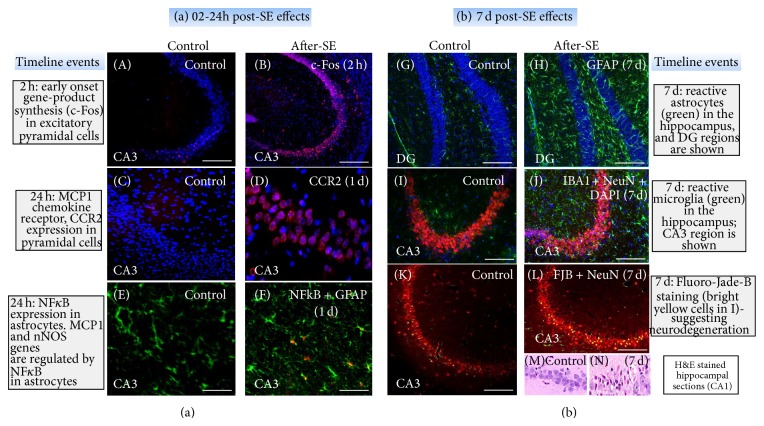
Immunohistochemistry (IHC) of the brain sections from kainate mouse and rat models of epilepsy at 2 h, 24 h, and 7 days after SE. (a) c-Fos ((A), (B)) expression was more widespread in the hippocampal formation at 2 h after SE (B). More than 3-4-fold increased expression (quantified data not shown) of c-Fos in CA3 pyramidal cell layer was observed (B). CCR2 ((C), (D)) and astrocytic NF*κ*B expression ((F), orange) at 24 hours after SE. (b) By 7 days after SE, there was increased astrogliosis ((H), GFAP, green) and microgliosis ((J), IB1A is marker for microglia, green) compared to controls ((G), (I)). SE induced neurodegeneration (FJB +ve neurons) was observed in CA3 of hippocampus (L). There were increased FJB +ve cells in CA3 of hippocampus (green label in (L), all scale bars 100 *μ*m). The same area was invaded by reactive astrocytes and microglia (green cells in (H) and (J)). Hematoxylin and Eosin stained hippocampal sections ((M), (N)) with pyknotic nucleus and shrunken cytoplasm are evident due to SE-induced changes at 7 days post-SE.

The period of epileptogenesis (latent period) follows immediately after an initial insult from the seizures. There is a transient increase in glutamine synthetase enzyme during this period in nonreactive astrocytes, which converts excess glutamate to inactive glutamine in the thalamus of human epileptic patients [108]. During the later course, glutamine synthetase activity gets downregulated in reactive astrocytes. As a result, excessive amount of nonmetabolized glutamate is released from astrocytes which accumulate in the extracellular space [125–129]. This results in hyperexcitability of neurons and later an onset of SRS. Astrocyte mediated glutamate release is discussed in later paragraphs. Although H_2_O_2_ increases during SE, it later decreases during the latent period due to activation of antioxidant systems [85, 130]. As the neuronal excitability increases, the SRS develops, which results in time dependent increase in H_2_O_2_ due to gradual depletion of antioxidant systems (GSH, coenzyme-A-SH) [131, 132]. As a consequence, accumulation of oxidized form of antioxidant enzymes, namely, GSSG and coenzyme-A-SSG, increases in the brain. A steady increase in ROS causes mitochondrial DNA damage resulting in downregulation of mitochondrial enzyme synthesis that is required for oxidative phosphorylation. Further electron transport chain complexes (1, 3, and 4) are affected [82, 111, 112]. The ROS also modifies the proteins subunits of excitatory ion channels and inactivates the energy-dependent glutamate transporters contributing to a further increase in neuronal hyperexcitability [71, 83]. An increase in ROS production is also contributed to a decrease in SOD and aconitase activity [48] (Figures 2 and 3). The neuronal hyperexcitability is further compounded by the loss of inhibitory GABAergic neuron populations of hippocampus and dentate gyrus leading to increased seizure susceptibility (Figures 4 and 5) [106, 133].

## 5. Mitochondrial Dysfunction and Lipid Peroxidation in TLE

The brain, being an organ with a low tolerance for hypoxic conditions due to neuronal need for oxygen, is particularly susceptible to ROS/RNS changes in mitochondria. Mitochondrial degeneration affects the stability of nuclear DNA (leading to chromosomal alterations), RNA, proteins, and lipids of the cell and also leads to defective calcium and glutamate homeostasis [41]. This increases the modulation of neuronal excitability and the synaptic transmission, an underlying mechanism in seizure production [84]. Waldbaum and colleagues investigated the changes that occur in the brain during the latency period that leads to the development of epilepsy [130]. Mitochondrial DNA gets repaired soon after the acute brain insult as a defensive mechanism; however this could be prolonged if the production of ROS/RNS during the insult is high. The concentration of H_2_O_2_ returns back to the basal levels during latency period but the production of ROS and RNS continues leading to the development of SRS [130]. It has also been suggested that certain protective enzymes, antioxidants, and coenzymes may be permanently damaged during this process [83, 113, 134, 135]. Furthermore, changes in DNA/RNA structure, compromised glutamate and calcium homeostasis, and depletion of antioxidant defense mechanism could lead to epileptogenesis [81, 85, 100]. According to Waldbaum and Patel, these changes affect all age groups. These disorders are most prevalent in the older people due to a reduced activity of antioxidant system which leads to the accumulation of free radicals resulting in neurodegeneration [117]. Waldbaum and Patel further proposed that ROS-induced mitochondrial DNA damage and decreased function of the electron transport chain are the major detrimental factors of neuronal death [136]. Oxidative stress leading to mitochondrial DNA alterations is also documented in patients with myoclonic epilepsy [137, 138].

Several hours after SE, the aconitase enzyme levels were found to reduce in mitochondria. Aconitase [an iron-sulphur protein] converts citrate into isocitrate in the TCA cycle. As TCA gets affected, the production of NADPH, flavin adenine dinucleotide (FADH_2_), and ATP reduces, which contributes to the development of SRS [48].

Lipid peroxidation, in general, is the conversion of fatty acids in the lipid bilayer to reactive species, resulting in neurodegeneration. As described earlier, polyunsaturated fatty acids are also present in large amounts within the inner membrane matrix of the mitochondria and are especially susceptible to lipid peroxidation by generating ROS [4]. Lipid peroxidation affects the permeability of the membrane, calcium pump activity, and most of the membrane bound enzymes [26, 92]—this is repeated. Studies revealed increased malondialdehyde (MDA) (measured as thiobarbituric acid reactive substances, TBARS) and F_2_-isoprostane levels that are derived from arachidonic acid cycle demonstrating that the lipid peroxidation indeed occurs during seizures [49, 139, 140]. Hydroxyl radicals that produce lipid peroxidation have also been found in the brains of rodent models of epilepsy [26, 93, 114, 116].

## 6. Role of Glia during Inflammation and Epileptogenesis

Gliosis (astrogliosis and microgliosis) occurs as a response to brain injury, which is characterized by proliferation and hypertrophy of the glial cells. Representative brain sections from 7 days after SE that were immunostained with glial markers are shown in Figures 4 and 5. Gliosis leads to formation of glial scar around the neurons that are under oxidative stress. Gliosis has both beneficial and detrimental consequences, which depends on their reactive state [141, 142].

### 6.1. Role of Astrogliosis

Astrocytes are the important source of antioxidants (neurotrophins) in the central nervous system (CNS) and play key role in cellular defense mechanism. Their protective role is regulated by nuclear factor erythroid related factor 2 (Nrf2), a transcription factor that mediates the production of antioxidants [143]. The Nrf2 activation is responsible for the regulation of antioxidant enzymes such as SOD, CAT, glutathione peroxidase (GSH-Px), and reduced form of GSH (GSHred). Astrocytes also play important roles in maintaining potassium homeostasis; glutamate uptake and release; lining of the blood brain barrier (BBB); providing nutritional, structural, trophic, and metabolic support to neurons; modulating synaptic activity; free radical scavenging; water transport and production of cytokines and NO [144]. Nonreactive astrocytes have also been found to play a neuroprotective role in recovering the neurons from brain injury by releasing trophic factors. The trophic factors include nerve growth factors, fibroblast growth factors, transforming growth factor-*β*, platelet-derived growth factor, brain-derived growth factor, and ciliary neurotrophic factor [141, 144]. All these factors play a part in stimulating neurite growth [144–147] and also promote angiogenesis, in case of cerebral ischemia, by expressing neuropilin-1 [144, 148].

Astrocytic glutamate transporters and neuronal glutamate receptors are known to play an important role in the pathogenesis of epilepsy. In normal brain, glutamate is taken up via astrocyte glutamate transporters from the extracellular space and metabolized to inactive glutamine to prevent excessive excitatory effects on neurons. Eid and coworkers have also shown a defect in the glutamine-glutamate cycle in hippocampal sclerosis patients that contributes to epileptogenesis [127, 129, 149]. Any alteration in this cycle is deleterious and can contribute to the hyperexcitability of neurons [150]. During the seizure insult, these astrocytic glutamate transporters become dysfunctional and lead to massive accumulation glutamate in astrocytes. This results in a release astrocytic glutamate (due to impaired astrocyte glutamate metabolism) into the extracellular spaces through a calcium dependent mechanism [150, 151]. This astrocytic glutamate release is also thought to be involved in amplifying the excitotoxicity of neurons [142, 152, 153]. The role of astrocytic glutamate in epileptogenesis has been debated for some time. However, it is largely agreed that the synaptic modulation by reactive astrocytes is one of the many causes of SRS [154]. Decreased expression and/or dysfunctional glutamate transporters in astrocytes, GLT-1 and GLAST, have been shown to be one of the key factors of human epilepsy [155].

The astrocytes become reactive, after the first seizure, due to changes at genetic, molecular, and cellular levels [141, 156]. A majority of these changes are observed at the transporter level in TLE during hippocampal sclerosis [157, 158]. In a normal astrocyte, the amount of glial fibrillary acidic protein (GFAP) was low as revealed by immunohistochemistry (Figure 5). Seven days after SE, GFAP was overexpressed, a hallmark of reactive astrocytes [159–161] (Figure 5). These reactive astrocytes secrete cytokines and chemokines such as IL-1B, tumor necrosis factor (TNF-alpha), interleukins (IL-1, IL-6, IL-10), and interferons (IFN-*α*, IFN-*β*) [162, 163], and chemoattractant protein-1 (MCP1). In addition to these factors, MCP1 may increase the calcium mediated glutamate release to worsen the epileptic state by producing hyperexcitability and a further production of ROS/RNS [164–167]. However, it is difficult to predict the effects of individual cytokine in reactive astrogliosis, as we can observe only net combined effects of all the cytokines in* in vivo* models. The cytokines are also known to produce pleiotropic effects. For example, excessive production of IL-6 and TNF-*α* promotes demyelination, thrombosis, leukocyte infiltration, and BBB disruption [162, 163], while under normal conditions IL-6 and TNF-alpha have neuroprotective effects in ischemic injury and excitotoxic injury models [168, 169]. Hence, the specific contribution of astrocyte cytokine release to the processes involved in the development of epilepsy remains to be established. Perhaps, the role of astrocytes changes at different stages of epileptogenesis.

### 6.2. Role of Microgliosis

Under normal conditions, microglia cells play a beneficial role to engulf the cellular debris and prevent cellular toxicity from spreading to the bystander neurons and also to recruit distant microglia to the site of injury. The inactive or ramified microglia has a small cell body with thin and slender branches. Activated microglia shows different morphology at different stages of activation [170]. Generally activated microglia, by 7 days after SE, has large cell body with/without thick projections/branches (Figure 4). At the time of injury or during excitotoxicity, these ramified microglia become active/reactive and undergo morphological changes [171–174]. However, during the early stages of insult, microglia are involved in neuroprotection and neurogenesis by releasing neurotropic and anti-inflammatory molecules [175]. Nonreactive microglia secrete neuroprotective factors such as brain derived neurotropic factor (BDNF) and NGF [176–180] and thrombospondin [174]. Microglia are mobile; they move to the site of injury and secrete proinflammatory cytokines and upregulate the expression of cell-surface molecules and membrane proteins [181, 182]. On the other hand when microglial cells becomes reactive, they can activate several inflammatory pathways/cyclooxygenase-2 (COX-2), interleukin (IL)-3, IL-6, Il-1B, tumor necrosis factor alpha (TNF-*α*), prostaglandins, tissue plasminogen activator (tPA), MCP-1, vascular endothelial growth factors, lymphotoxin, matrix metalloproteinases, and macrophage inflammatory protein-1*α* [172–178]. The amount of secretion of such factors depends upon the severity of the insult. For example, activation of tPA, along with other factors, has been shown to play a role in the mossy fiber sprouting (MFS) which is observed in chronic epilepsy [183–186]. Further, an increased number of activated microglia near the damaged tissues [36, 187], especially at CA1 and CA3 regions of the hippocampus, prove their harmful role during epilepsy (Figure 5). It has also been proposed that microglial activation can sustain the development of SRS by initiating aberrant neurogenesis and also the migration of neuroblasts in the dentate gyrus [188]. Our ongoing work demonstrates increased expression of chemokine receptor 2 (CCR2), the receptor for MCP-1 (Figure 5). Incidentally, MCP-1 production by astrocytes is mediated through nuclear factor kappa-light-chain-enhancer of activated B cells (NF-kB) and was also found upregulated following 24 h after SE (Figure 5).

### 6.3. Crosstalk between Neuron and Glia during Epileptogenesis

Seizures during the SE subsequent spiking activity and repeated SRS activate the resident glial cells (astrocytes and microglia) to become hypertrophic and reactive (Figures 4 and 5). As discussed above, the reactive glial cells release proinflammatory mediators which in turn act on the neurons to decrease their seizure threshold. There is also increased expression of redox-sensitive transcription factors activator protein-1 (AP-1) and NF*κ*B leading to an activation of NADPH oxidase in microglia cells. The activation of NADPH oxidase on microglia cells results in the formation of cytochrome b_558_ in the electron transport chain, which leads to an increase in extracellular superoxide production through iNOS. These factors may affect neuroblasts and/or those neurons that were recovering during post-SE phase of epileptogenesis (Figure 4).

Overall, the chain of events that occur following seizures is summarized below; (a) increase in intracellular calcium due to activation of NMDAR during and soon after SE or seizure (illustrated in Figure 4); (b) activation of phospholipase A_2_ (arachidonate release) and phospholipase C (not shown); (c) immediate early gene expression such as c-Fos; (d) altered kinase activity, altered phosphorylation of enzymes, receptors, and ion channels (not shown); (e) altered ion channel function as evident from increase spiking activity; (f) change in subunit expression of excitatory and inhibitory receptors; (g) altered synaptic morphology, remodelled dendritic spines; (h) enhanced neurogenesis in dentate gyrus; (i) MFS leading to altered connectivity; (j) oxidative damage to proteins, lipids, and DNA; and (k) neurodegeneration through apoptosis inducing factor (AIF) or caspase-3 mediated pathway (illustrated in Figure 4). The emerging hypothesis in our laboratory is that targeting the postsynaptic membrane proteins could be protected against the recurrence of seizures. The postsynaptic density protein-95 (PSD-95), a scaffolding protein that links the nNOS with glutamate receptors, is depicted in Figure 4. Modulating protein-protein interactions involved in disease pathways is an attractive strategy for developing drugs but remains a challenge though. One approach is to target certain domains within proteins that mediate these interactions. One example of such a domain is the PDZ domain of PSD-95, which is involved in interactions between many different proteins in a variety of cellular contexts. Because PDZ domains have well-defined binding sites, they are promising targets for drug discovery in epilepsy research.

## 7. Treatment Options for TLE

### 7.1. Antiepileptic Drugs: Beneficial and Adverse Effects

Several AEDs have been tried for TLE. AEDs used to suppress seizures in epileptic patients have multiple mechanisms of action [189]. For example, phenytoin reduces the amplitude of sodium channels by inactivating them; ethosuximide blocks Ca^2+^ channels; phenobarbital blocks GABA_A_ receptors and possibly sodium channels; and carbamazepine (CBZ) prevents convulsions by potentiating certain GABA receptors subtype containing *α*1, *β*2, and *γ*2 subunits [190]. A long term use of AEDs leading to impairment of the endogenous antioxidant system has been investigated. AEDs, namely, valproic acid, phenytoin, CBZ, and levetiracetam, are shown to increase lipid peroxidation and decrease GSH/GSH-Px [4]. CBZ is implicated in reduction of CAT enzyme activity while phenobarbital valproic acid (VPA) and CBZ are shown to reduce SOD enzyme activity [4]. In rat cortical astrocyte cell culture assays CBZ, oxcarbazepine, and topiramate are demonstrated to cause oxidative stress leading to reduced activity of astrocyte glutamine synthetase [191, 192]. Phenobarbital, CBZ, and valproic acid after their hepatic metabolism result in reactive intermediates that can lead to covalent binding to macromolecules [193, 194]. From our ongoing proteomics studies from 7 days post-SE mouse model (kainate), we have identified downregulation of VPA transporter protein. VPA is a broad spectrum AED and one of the most widely prescribed drugs for epilepsy worldwide. Its effects are mediated by an action on the inhibitory system, *γ*-aminobutyric acid (GABA), through enhancement of GABA synthesis and release [195]. VPA is also histone deacetylase (HDAC) inhibitor and has a neuroprotective role [196]. A review by Cárdenas-Rodríguez et al. summarizes the effects of AEDs on the markers of oxidative stress in human epileptic patients [197]. Although AEDs control seizures, their role to elicit systemic toxicity and to contribute to oxidative stress needs to be carefully considered during therapy. Moreover, since AEDs only control symptomatic seizures, an adjunct therapy such as dietary supplements and neuroprotectants would be beneficial. In this review, the role of dietary supplements in epilepsy is briefly discussed below.

### 7.2. Role of Enzymatic and Nonenzymatic Antioxidants

The cells possess endogenous antioxidant system to neutralize and scavenge free radicals when produced in excessive amounts. As explained earlier in this paper, these scavengers are enzymes such as SODs, catalases, glutathione/glutathione peroxidase system, and thioredoxin reductases. The other nonenzymatic antioxidant systems include cysteine/glutamate antiporter and dietary supplements such as vitamins E and C, polyphenols, melatonin, and ketogenic diet.

### 7.3. Glutathione and Cysteine/Glutamate Antiporter

GSH has been found to be low in epileptic patients by about 150% when compared to nonepileptic patients [131]. Reduced glutathione, a tripeptide with a free sulfhydryl group, is required to combat oxidative stress and to maintain homeostasis in the cell. Selenium (Se) acts as a catalyst for GSH-Px activity and has similarly been studied in children with epilepsy. It has been found that blood serum Se concentrations are lower in epileptic children than healthy children [198, 199]. But low levels of selenium detected in epileptic patients did not exhibit typical signs of selenium deficiency such as generalized fatigue, light sensitivity, and heart palpitations [193].

Cysteine/glutamate antiporter system (CGS) is a protective antioxidant mechanism. The neurons exchange intracellular excitotoxic glutamate for oxidized cysteine from the extracellular space. GCS is found in both neurons and glial cells (astrocytes, microglia) in the brain [200–204]. Glutamate exported by CGS is responsible for the extracellular glutamate concentration in the brain which is later taken up astrocytes to be converted into inactive glutamine. The oxidized cysteine imported into the cell is essential for the synthesis GSH by enzyme thioredoxin reductase 1. Thus CGS acts like a bridge that connects the antioxidant defense with neuronal excitability. The CGS system gets impaired during an increased extracellular glutamate (during astrocytic glutamate release) and/low intracellular cysteine. Thus an increase in extracellular glutamate apart from inhibiting CGS is also responsible for hyperexcitability of neurons. Thus an inhibition of CGS can lead to depletion of endogenous glutathione reserves succumbing to oxidative stress and cell death termed as “oxidative glutamate toxicity.” Impaired CGS system has also been implicated in other neurological disorders apart from epilepsy [205–209]. Therefore, the drugs that enhance CGS can be beneficial.

### 7.4. Antioxidant Diet Supplements

(i)* Vitamin C.* Vitamin C, due to its water soluble nature, was found to be effective in eliminating free radicals within the brain circulation. The recommended dietary allowance (RDA) for vitamin C is 75–90 mg/day for adults. Red peppers, oranges, grape fruits, and kiwi fruits are the rich sources of vitamin C [210]. In rat models of epilepsy, pilocarpine increased lipid peroxidation during SE. Vitamin C caused a decrease in lipid peroxidation and increase in CAT enzyme activity. Further, vitamin C also increased the latency to the onset of seizures after SE while reducing the mortality rates in rat models [211]. 

(ii)* Vitamin E*. Vitamin E was found to exert its anticonvulsive effects by upregulating catalase activity in pilocarpine rodent models of epilepsy [123, 124, 211]. The RDA for vitamin E is 15 mg/day (22.4 IU) for adults. Wheat germ oil, sunflower seeds, almonds, and hazelnuts are the rich sources of vitamin E [210]. During pilocarpine induced seizures, vitamin E concentrations were found to decrease in brain cortex [212]. Frantseva and colleagues in kindling rat models of epilepsy showed that antioxidant treatment (vitamin E and glutathione) reduced neuronal death and lipid peroxidation; however, it did not prevent development of recurrent seizures [53]. 

(iii)* Polyphenols.* Cloves, peppermint, cocoa, oregano, flaxseeds, and chestnuts are the rich sources of polyphenols [213]. Food groups such as polyphenols derived from commercial and organic grape juice and yerba mate have been demonstrated to prevent neurodegeneration and seizures [214, 215]. Branco and colleagues have found that organic yerba mate is found to reduce seizures by increasing SOD and CAT activity in rodent models [215]. 

(iv)* Melatonin*. Melatonin has been found to act as scavenger of hydroxyl radicals to prevent lipid peroxidation in the CNS [216]. Melatonin rich plant sources include St. John's wort, fennel seed, sunflower seed, fenugreek seed, and black mustard seed [217, 218]. Although not approved by FDA, 0.3–5 mg/day for an adult was found to be beneficial in sleep disorders [219]. Since melatonin has both lipophilic and hydrophilic properties, it is speculated that it could be an effective antioxidant. In a mice study, when melatonin was given concurrently or 30 min prior to induction of seizure with kainate, it attenuated the lipid peroxidation [122]. Interestingly, in the same study melatonin when given 15 minutes after SE had no effect on the seizure suggesting that a high level of melatonin prior to seizure induction has beneficial effects. 

(v)* Ketogenic Diet*. A typical ketogenic diet is a high-fat, low carbohydrate diet containing long chain fatty acids providing 3-4 grams of fat for every gram of carbohydrate and protein [220]. The ketogenic diet has been demonstrated to reduce mitochondrial ROS/RNS due to a change in source of energy using fewer carbohydrates and more fat-derived ketone bodies [221–224]. This protective effect can be traced to high acetone concentrations present in the brains of human patients on ketogenic diet. Ketogenic diet demonstrated anticonvulsive effects in epileptic children with congenital abnormalities such as mutations glutamate transporter, GLUT-1 [225], and a deficiency of pyruvate dehydrogenase [223, 226]. High-fat diet was found to initiate epilepsy in infant mice that lack mitochondrial uncoupling protein (UCP) isoforms and this effect was neutralized by a low-fat diet [227, 228]. These data infer the protective effects of a high-fat diet during epileptic seizures, however, the age of the individual being an important criterion.

### 7.5. NOS Inhibitors to Prevent Epileptogenesis

NOS inhibitors such as N-propyl-L-arginine (L-NPA) and nitro-L-arginine methylester (L-NAME) have been tested in experimental rodent models of epilepsy [31, 36, 229–233]. L-NPA, a selective nNOS inhibitor, reduced the frequency of epileptiform spikes, severity, and duration of seizures during 7 days after SE in kainate mouse (C57BL/6J) model of epilepsy [36]. Studies showed that a broad spectrum NOS inhibitor, L-NAME, had a controversial role on hippocampal damage or protection in rat models (quote our papers from Siobhan and 200), while aminoguanidine selective iNOS inhibitor significantly reduced seizures in a kainate mouse model of epilepsy [229]. Another potent and highly selective inhibitor of iNOS, 1400 W, has been studied for its effects on inhibiting iNOS in both* in vivo* and* in vitro* models [87, 234]. 1400 W is a slow, tight binding, and a highly selective pharmacological inhibitor of human iNOS with a dissociation constant (Kd) value of 7 nM and a selectivity of 5,000-fold for iNOS [30, 234]. Due to its selective action, 1400 W is found to have little or no cardiovascular side effects and does not interfere with the physiological activities mediated by nNOS [234]. 1400 W was found to be most effective during pathological increase in iNOS levels in various organs [30, 234–236]. 1400 W is BBB permeable and biologically active* in vivo* and effective in ameliorating the neuropathological changes in traumatic brain injury and stroke models by decreasing glutamate release [237–239]. Additional advantage of iNOS inhibitors is that they attenuate BBB leakage [240]. Serum albumin (SA) is considered as a biomarker for BBB leakage [241, 242]. Recent studies in the hippocampus suggest that increased SA levels are responsible for hyperexcitability of neurons and SRS due induction of reactive astrogliosis as validated by increased GFAP levels [240, 242]. Our recent proteomics studies of hippocampus from 7 days post-SE mice provide evidence for concomitant increased levels of both SA and GFAP. In those studies, 1400 W reduced SA and GFAP to their basal levels (63-64). From our ongoing work, immunohistochemistry of brain sections from 7 days after SE in an organophosphate rat model revealed an important polarizing effect of 1400 W on gliosis. It decreased reactive microglia, which could be due to decreased levels of glutamate and SA, but increased the number of nonreactive glial cells (data not shown). A recent article highlights the therapeutic importance of drugs that polarize glial cells from reactive to nonreactive state [243]. Nonreactive gliosis is neuroprotective [244–247]. Our EEG analyses from 1400 W treated rats at 7 days after SE confirmed a decrease in spike rate when compared to vehicle treated in a diisopropyl fluorophosphate (DFP) model suggesting that 1400 W decreases neuronal hyperexcitability by reducing proinflammatory cytokines and by promoting neurotropic activity. Although 1400 W is a highly specific iNOS inhibitor and is emerging as a promising disease modifying drug for epilepsy, its mechanism of action is not yet clear. It has been found that 1400 W was able to reduce phosphorylation of c-Jun N-terminal kinase (JNK), but it was unable to prevent seizures from occurring [229, 234], possibly due to inappropriate dosing regimen. JNK acts as a signaling molecule during stress, such as UV radiation and oxidative stress and phosphorylation. JNK is also responsible for neurodegeneration and apoptosis [38]. Hence, an inhibition of JNK prevents neurodegeneration [248, 249] and may offer antiepileptic therapeutic option by iNOS inhibitors.

## 8. Conclusion

In summary, oxidative stress plays a key role in epileptogenesis after the first seizure. Through progressive neurobiological changes, the first seizure later becomes a cause for recurrent seizures in TLE. The acute effect of oxidative stress is neurodegeneration, which is mediated by seizure-induced reactive gliosis. Oxidative stress targets mitochondrial DNA and lipid peroxidation which affect ATP depletion and further contributes to excessive production of ROS/RNS. These changes override endogenous antioxidant protective mechanisms. These changes will induce rearrangement neural circuits, neuronal loss and neurogenesis, and aberrant migration of neuroblasts thus contributing to hyperexcitability and SRS onset. Breaking this vicious cycle is critical by developing new and effective drugs which can prevent epileptogenesis. The current AEDs in combination with neuroprotectants and/or antioxidants could be effective in disrupting the vicious cycle. Role of antioxidant supplements, ketogenic diet, COX-2 inhibitors, NOS inhibitors, and PSD-95 blocking peptide are some of the options currently being explored to complement existing AEDs to control epilepsy. Initial success of these treatment options in different animal models and some human patients is encouraging. However, intense investigation is required to fully evaluate the potential of a combination of drugs to cure established epilepsy and refractory epilepsy.

## Figures and Tables

**(a):** 

Rat kainate model	4 hr	8 hr	16 hr	24 hr	48 hr	3–7 d	3 week	Human patients	
GSH/GSSG ratio	↑			↑				GSH/GSSG ratio	↓
Lipid peroxidation (TBA assay)	↑	↑	↑	↑	↓	↓	↓	Lipid peroxidation (TBA assay)	↑
Protein oxidation	↑			↑				Protein oxidation	↑
SOD					↑		↑	SOD	↑
NADPH oxidase						↑	↑	catalase	↑
catalase					↑			aconitase	
aconitase		↓	↓		↓			DNA damage (OdG assay)	↑
DNA damage (OHdG assay)			↑	↑	↑				

**(b):** 

Rat kindling model	4 hr	8 hr	16 hr	24 hr	48 hr	3–7 d	3 week
GSH/GSSG ratio			↓	↓	↓		
Lipid peroxidation (TBA assay)				↑			↑
SOD				↓			
aconitase			↓		↓		

**(c):** 

Rat Pilocarpine model	4 hr	8 hr	16 hr	24 hr	48 hr	3–7 d	3 week
GSH/GSSG ratio				↓			
Lipid peroxidation (TBA assay)	↑	↑		↑	↑	↑	
SOD	↑	↑		↑	↑	↑	
catalase				↑			

**(d):** 

Mice kainic acid model	4 hr	8 hr	16 hr	24 hr	48 hr	3–7 d	3 week
GSH/GSSG ratio					↓		
Lipid peroxidation (TBA assay)				↑	↑		↓

Rat kainate model [48–52], Rat kindling model [53–57], Rat pilocarpine model [58–60], Mouse kainate model [61–64], Human patients [65–67].
